# Meetings of Societies

**Published:** 1916-07

**Authors:** 


### MEETINGS OF SOCIETIES.

### Bristol Medico-Chirurgical Society.

*May 10th*, 1916.

Major George Parker, President, in the Chair.

Captain J. M. Fortescue-Brickdale read **Notes on the Enteric Group of Diseases as seen in an Isolation Hospital in France.** (See p. 73.)

Captain E. W. Hey Groves read a paper entitled **Brains, Bones and Blood-vessels,** being Notes on some Surgical Experiences in the Near-East during the present War.

